# Assessment of the Biocompatibility of Cucurbiturils in Blood Cells

**DOI:** 10.3390/nano11061356

**Published:** 2021-05-21

**Authors:** Alina Aktanova, Tatjana Abramova, Ekaterina Pashkina, Olga Boeva, Lyubov Grishina, Ekaterina Kovalenko, Vladimir Kozlov

**Affiliations:** 1 Research Institute of Fundamental and Clinical Immunology, 6300099 Novosibirsk, Russia; aktanova_al@mail.ru (A.A.); tatjana-abramova@mail.ru (T.A.); starchenkova97@gmail.com (O.B.); l_grishina@bk.ru (L.G.); vakoz40@yandex.ru (V.K.); 2 Department of Medicine, Novosibirsk State University, 630090 Novosibirsk, Russia; 3 Nikolaev Institute of Inorganic Chemistry SB RAS, 630090 Novosibirsk, Russia; e.a.kovalenko@niic.nsc.ru

**Keywords:** nanoparticles, cucurbiturils, cytotoxicity, immunosafety, apoptosis, hemolysis

## Abstract

Currently, cucurbiturils are being actively researched all over the world. Research is focused on the ways of improving the solubility and selectivity of cucurbiturils, increasing the stability of the complexes with other particles in various media and enhancing their ability to bind and release various substances. The most significant area of our research is the assessment of safety, studying the biological properties and synergistic effects of cucurbiturils during complexation with drugs. In this article, the hemocompatibility of erythrocytes and leukocytes with cucurbiturils was investigated. We demonstrated that cucurbiturils have no cytotoxic effect, even at high concentrations (1 mM) and do not affect the viability of PBMCs. However, cucurbiturils can increase the level of the early apoptosis of lymphocytes and cucurbit[7]uril enhances hemolysis in biologically relevant media. Despite this, cucurbiturils are fairly safe organic molecules in concentrations up to 0.3 mM. Thus, we believe that it will become possible to use polymer nanostructures as drug delivery systems in clinical practice, since cucurbiturils can be modified to improve pharmacological properties.

## 1. Introduction

Cucurbit[n]urils (CB[n]s) are macrocyclic cavitands containing glycoluril monomers (n) linked by methylene bridges. Robert Behrend synthesized CB[n] in 1905 by a condensation reaction of urea, glyoxal and formaldehyde in an acidic medium [1]. All homologues of cucurbiturils have similar structural features: monomeric glycoluril units with highly polarized oxygen atoms in carbonyl groups (known as a “portal”), which are associated in a closed hydrophobic cavity with an increasing size (from 5.8 Å for CB[6] to 8.8 Å for CB[8]) and a constant height (9.1 Å) [2]. The internal and portal dimensions of cucurbiturils lead to specific molecular recognition behavior, so they unequally cooperate with organic molecules. Cucurbiturils, ranging from CB[6], encapsulate molecules according to the guest–host type on the formation of inclusion complexes with unique physicochemical and, accordingly, biological properties. Currently, antitumor, antibacterial and anticholinergic medicines, antioxidants, neurotransmitters, cholinesterase reactivators, etc., are placed into the cucurbituril cavity. For example, CB[7] is able to encapsulate oxaliplatin [3], cisplatin [4], methotrexate [5], paclitaxel [6], ambroxol hydrochloride [7], clofazimine [8] and oxime K027 [9]. In turn, CB[8] forms complexes with oroxin A [10], mitoxantrone [11] and polyethyleneimine [12]. These articles reported on the formation of a stable inclusion complex in ratios of either 1:1 or 2:1, the possibility of using higher dosages of these medicinal substances, increasing efficiency and reducing toxicity. These data assert and allow using cucurbiturils as a remarkable drug delivery system. Special cases of the encapsulation by cucurbiturils of serotonin [13] and histamine H2 antagonists such as cimetidine, famotidine and nizatidine [14] in the 1:1 inclusion complex allow to expand the possibility of cucurbituril’s application for various nosologies. Recently, it was reported that complexation CB[7] with chloroquine permits reducing its nonspecific toxicity over several rounds and improves its antiviral activity against coronavirus, confirming the pronounced synergistic effect of cucurbiturils. CB[7] has also been found to form 1:1 host–guest complexes with chloroquine [15]. Regarding nanomaterials, their toxicity depends on the determinants, which include exposure time, dose, aggregation and concentration, particle size and shape and surface area and charge. Depending on the size and shape, even among the homologues of the same compound, different physicochemical and, accordingly, biological properties can be demonstrated.

However, despite the fact that there is a great amount of data about cucurbiturils, their biological compatibility has not been well studied. A comprehensive assessment of selective toxicity is required to analyze its risks as a medicinal agent. One of the properties of medicinal substances is hematotoxicity—a type of selective damage of the function of blood cells or their cellular component. The most significant functions of blood cells are oxygen transport, hemostatic and immunity provision. Hemolysis of erythrocytes occurs due to their dysfunction, cell death through direct damage to the cytoplasmic membrane of cells, or the induction of cell apoptosis as a manifestation of hematotoxicity.

Apoptosis is a process of programmed cell death. It has been reported that nanoparticles are able to induce cell apoptosis due to various mechanisms, such as activation of mitochondrial pathways, increased expression of caspases 3, 8, 9 and other signaling pathways [16]. Cyclodextrin is a structural analog of cucurbituril, which induces apoptosis in human keratinocyte cells HaCaT [17] and the HepG2 cell line [18] due to activation of the caspase pathway. The cucurbit[8]uril contained in a complex polymer structure induces cell apoptosis in vivo [19]. In a recent study, Fink and colleagues reported that cucurbit[7]uril induces apoptosis in HaCaT human keratinocyte cells. The number of apoptotic cells depends on the concentration of CB[7]. A high concentration of CB[7] causes more than 50% of apoptotic cells. In this study, the effect of cucurbiturils on RBC was assessed, since the determination of the toxicity of cucurbiturils has been established for a small quantity of cell lines [20]. However, hemolysis was assessed only in PBS, while the components of a culture medium can influence this process. Cucurbiturils are known to interact with amino acids, peptides and proteins [21,22,23,24,25]. Serum contains a great number of proteins; the interaction of cucurbiturils with these proteins may be significant for hemolytic activity. The hemolytic properties of medicinal agents have to be investigated, because it is expected that CB[n] can enter the systemic circulation and affect human blood cells. It is also necessary to evaluate the cytotoxic effects of cucurbit[6,7,8]urils on leukocytes, which are represented by mononuclear cells.

## 2. Materials and Methods

Cucurbit[n]urils (n = 6, 7 and 8) were synthesized, purified and kindly provided by the Nikolaev Institute of Inorganic Chemistry (Novosibirsk, Russia). Before the experiments, CB[n] was diluted in the culture medium RPMI-1640. CB[6] and CB[7] were prepared at a concentration 0.3, 0.5 and 1 mM; CB[8] at 0.01 mM and in a PBS solution; CB[6] and CB[7] at 0.2, 0.5, 1 and 2 mM; CB[8] at 0.01 mM. RPMI-1640 (Biowest LLC, Riverside, MO, USA), gentamicin (Dalfarma, Khabarovsk, Russia), tienam (Merck Sharp & Dohme Corp., Kenilworth, NJ, USA), FCS (Hyclone, Chicago, IL, USA), tabs for preparing phosphate-buffered saline (PBS) (AppliChem GmbH, Darmstadt, Germany), Na2EDTA (Helicon, Moscow, Russia), Ficoll (BioClot, Aidenbach, Germany), urografin 76% (Schering, Berlin, Germany), human albumin solution (Microgen, Moscow, Russia), Triton^™^ X-100 (Dow Corning, Midland, MI, USA, WST-1 assay kit (Takara Bio, Kusatsu, Japan), LDH-Cytox™ Assay Kit (BioLegend, San Diego, CA, USA), PE Annexin V Apoptosis Detection Kit with 7ADD (BioLegend, San Diego, CA, USA), anti-CD3 antibody (BioLegend, San Diego, CA, USA) and anti-CD4 antibody (BioLegend, San Diego, CA, USA).

The Ethical Committee of RIFCI, Russia, approved the study design and the recruitment of subjects. Subjects provided written informed consent. The relevant guidelines and regulations were followed when performing the experiments.

### 2.1. Isolation of PBMC

Peripheral blood was obtained from 21 healthy volunteers (average age, 28.8 ± 0.39 years) after receiving their written consent. Peripheral blood mononuclear cells (PBMCs) were isolated by density-gradient on a Ficol-urografin (1.078). The PBMCs were washed twice with phosphate-buffered saline (PBS) and EDTA. The cells were counted in 3% acetic acid using a Goryaev chamber.

### 2.2. PBMC Cytotoxicity Assay

Isolated PBMCs at a concentration of 1 × 10^5^ cells/mL were cultured with CB[6], CB[7] and CB[8] in a culture medium RPMI-1640, supplemented with 50 mg/mL of gentamicin, 25 mg/mL of tienam and 10% FCS in a 96-well plate (TPP, Trasadingen, Switzerland) for 72 h in a humidified atmosphere of 5% CO_2_. CB[6] and CB[7] were added at concentrations of 0.3, 0.5 and 1 mM, while CB[8] was added at 0.01 mM due to the low solubility of this compound in mediums. The wells were duplicated. After cultivation cells were collected and washed with PBS, WST assay reagents were added to the cell culture media and then incubated for 4 h. The cytotoxicity of CB[n] was analyzed by the amount of formazan dye produced by measuring the absorbance at 450 nm.

PBMCs at a concentration of 1 × 10^5^ cells/mL were cultured with CB[6,7,8] at the same concentrations and conditions as the WST assay for 24 h to release LDH into the cell culture medium. To perform the assay for the cellular cytotoxicity of CB[n], the reaction mixture was added to the wells of the cell culture medium. After 30 min of incubation with lysis buffer and 30 min of incubation with assay buffer with protection from light, the reaction was stopped by adding a stop solution and the absorbance was measured using a microplate reader Infinite F50 (Tecan, Grödig, Austria) at 490 nm. The average absorbance of each triplicate set of wells was calculated and the background control value was subtracted. The percent cytotoxicity was calculated with the following equation:

Cytotoxicity (%) = ((A − C)/(B − C)) × 100(1)

A: Test Substance, B: High Control, C: Low Control.

### 2.3. Apoptosis of the PBMC Assay

The blood was obtained from 11 healthy volunteers without acute and exacerbated chronic diseases. The volunteers provided informed consent to perform the necessary manipulations.

In freshly isolated lymphocytes (CD3^+^ PE/cy7; CD4^+^ APC/cy7), the initial (native) levels of early (AnV^+^/7AAD^−^, %) and late (AnV^+^/7AAD^+^, %) apoptosis were determined. The lymphocytic fraction of the cells at a concentration of 5 × 10^5^ cells/mL was incubated with CB[6], CB[7] and CB[8] in the culture medium RPMI-1640 in 48-well plates (TPP, Trasadingen, Switzerland) for 72 h in a humidified atmosphere with 5% CO_2_. CB[6] and CB[7] were added at concentrations of 0.3 mM and 1 mM and CB[8] at 0.01 mM. After cultivation, the cells were collected and washed with PBS. The toxic effect of cucurbiturils on the population of T-lymphocytes (CD3^+^/CD4^+^; CD3^+^/CD4^−^) of healthy donors was established (on day 3 of incubation) according to the level of early (AnV^+^/7AAD^−^, %) and late (AnV^+^/7AAD^+^, %) apoptosis. The determination of apoptosis was performed in accordance with the instructions for a complex of immunological tests containing AnnexinV (AnV), labeled with fluorochrome-conjugated PE and 7AAD—or labeled with APC (Becton Dickenson, Franklin Lakes, NJ, USA) in the control—and incubated in vitro with various concentrations of the test substance. Samples were recorded and analyzed on a FACS Canto II flow cytometer APC (Becton Dickenson, Franklin Lakes, NJ, USA) using FACSDiva 6.1 software APC (Becton Dickenson, NJ, USA).

### 2.4. Hemolysis Assay

A group of volunteers (n = 8) was formed to determine hemolysis by CB[6,7,8]. The separation of erythrocytes from plasma was performed by 2.7 rpm centrifugation with ficoll (it provides the density gradient) for 25 min. After plasma removal, the erythrocytes were washed two times with PBS by centrifugation 800× *g* for 10 min. Then, the erythrocytes were suspended in PBS to obtain 2% hematocrit. The erythrocytes were used immediately.

Next, a 2% RBC suspension was treated with CB[6] and CB[7] (CB[n] diluted in PBS) at concentrations of 0.2, 0.5, 1 and 2 mM and CB[8] at 0.01 in PBS, albumin (40 g/L), or an autologous serum from donors. Every sample was incubated 1 h by a shaker in a thermostat at 37 °C. The positive control was treated with Triton-100 (10 *v*/*v*) and the negative control was included in RBC in different biologically relevant media (PBS, albumin, or serum). After incubation the samples were centrifuged to 3.5 rpm × 15 min. The supernatant was transferred to wells to measure the absorbance of release hemoglobin at 540 nm. The level of hemolysis was calculated using the formula:

Hemolysis (%) = 100 × (Abs − Ab_0_)/((Abs_100_ − Abs_0_))) (2)

Abs—absorption index for the test sample;

Ab_0_—absorbance index for negative control (corresponding buffer);

Abs_100_—Absorption index for positive control (Triton X-100).

### 2.5. Statistical Analysis

ANOVA analyses were performed using GraphPad Prism, with Friedman and Dunn’s multiple comparisons tests. A *p*-value < 0.05 was regarded as the minimum criterion for statistical significance.

## 3. Results

### 3.1. Cytotoxic Effect of Cucurbit[6,7,8]urils on PBMCs

**Figure 1:**
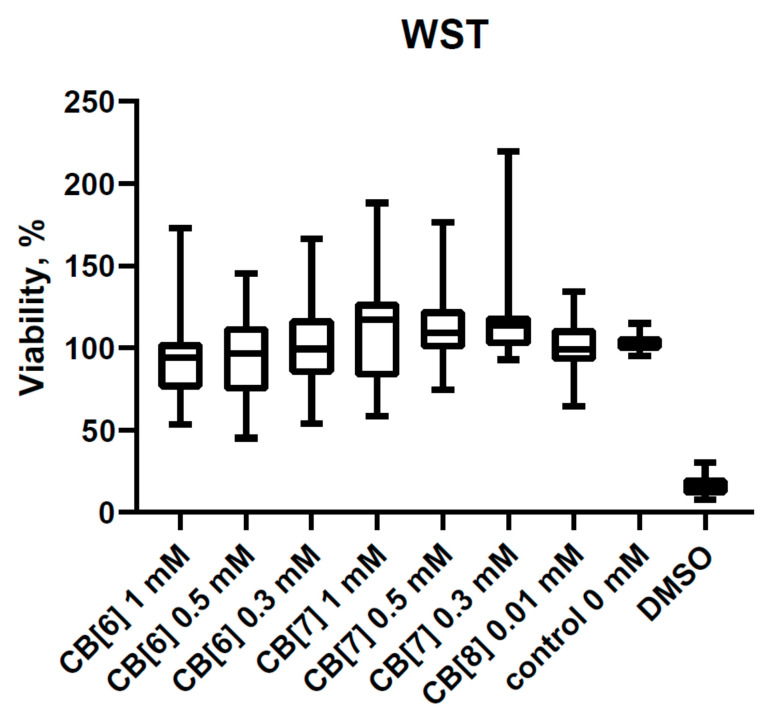
The viability of peripheral blood mononuclear cells (PBMCs) with or without cucurbit[n]uril (n = 6, 7, 8) at different concentrations. Data are presented as box-and-whisker plots, with boxes extending from the 25th to the 75th percentile, with a horizontal line at the median, while the whiskers extend to the lowest and highest data points (n = 13).

**Figure 2:**
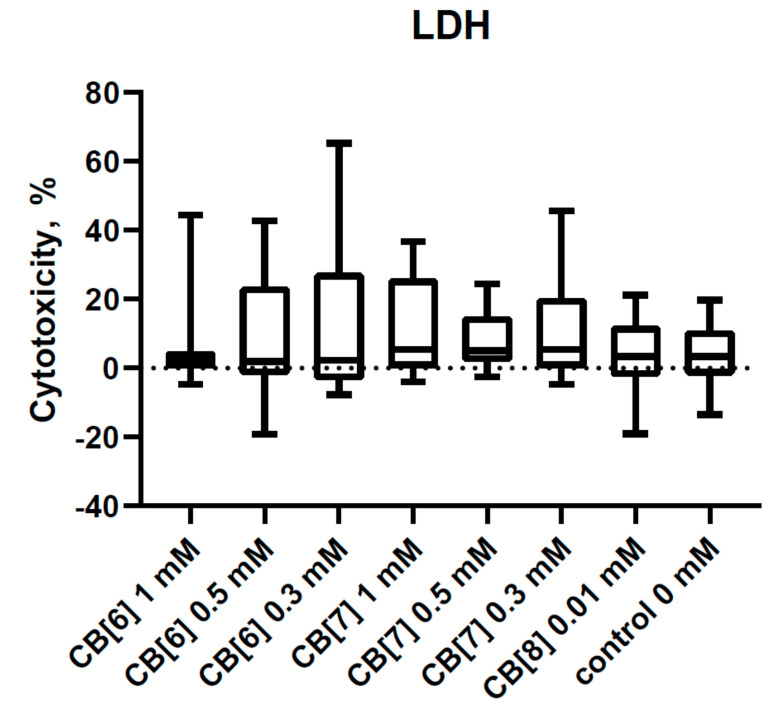
Lactate dehydrogenase (LDH) leakage in PBMCs after CB[6], CB[7], or CB[8] exposure. Data are presented as box-and-whisker plots, with boxes extending from the 25th to the 75th percentile, with a horizontal line at the median, while the whiskers extend to the lowest and highest data points (n = 13).

Recently, we described the effect of CB[6] and CB[7] on the viability of PBMCs [26]. It is already known that CB[6] and CB[7] do not reduce the viability of PBMCs at a concentration of 1 mM and below. In this study, we supplemented the known data with an assessment of the effect of CB[8] on the viability of PBMCs (Figure 1). The concentration at 0.01 mM was used due to CB[8] poor solubility in water and biologically relevant media (PBS and RPMI-1640). As is well known, the assessment of viability is associated with the metabolic activity of mitochondria, specifically with cellular mitochondrial dehydrogenase. The quantity of the dye was generated by activity of dehydrogenase, directly proportional to the amount of living cells. However, the production of dehydrogenases in PBMCs is sufficiently low, which may complicate the assessment of the percentage of viable PBMCs. Therefore, we additionally evaluated the cytotoxicity of CB[n] using the LDH assay test (Figure 2). This assay measures the release of cytoplasm enzyme LDH by damaged cells. No differences in LDH release were observed with the addition of CB[n]. Therefore, the cytotoxicity effects of the studied concentration of CB[n] are not shown.

### 3.2. Effect of Cucurbit[6,7,8]urils on the Apoptosis of PBMCs

**Figure 3:**
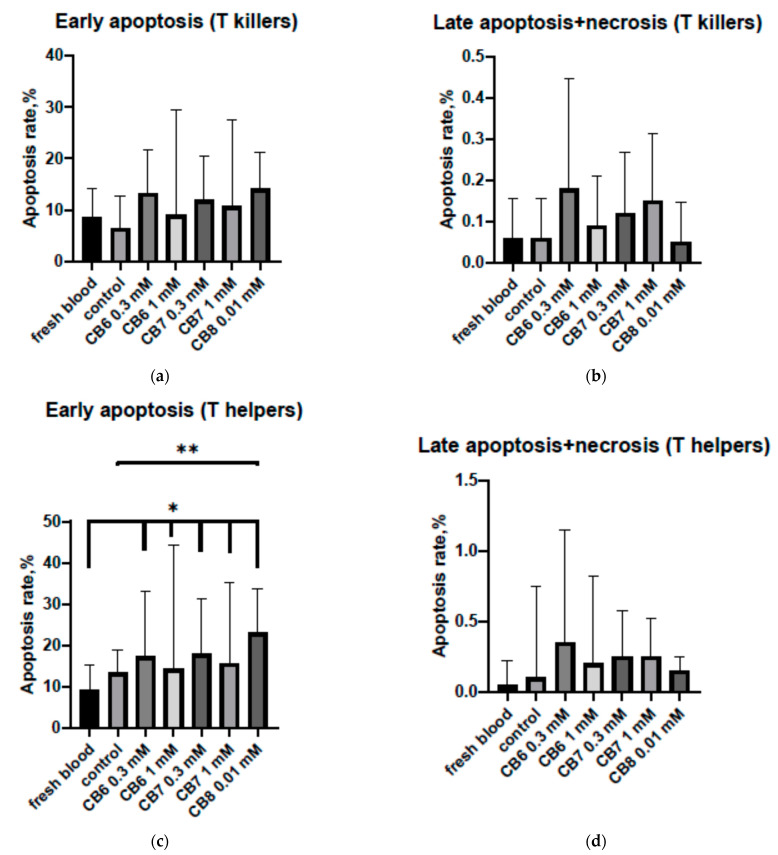
Effect of cucurbit[6,7,8]urils on the apoptosis of PBMCs: (**a**) Effect of cucurbit[6,7,8]urils on the early apoptosis of T killers; (**b**) effect of cucurbit[6,7,8]urils on the late apoptosis and necrosis of T killers; (**c**) effect of cucurbit[6,7,8]urils on the early apoptosis of T helpers; (**d**) effect of cucurbit[6,7,8]urils on the late apoptosis and necrosis of T helpers. * Indicates a significant difference (*p* < 0.05) vs. the apoptosis in fresh blood; ** indicates a significant difference (*p* < 0.05) vs. the control. Data are presented as the median with the interquartile range (n = 11).

The next stage was evaluating the effect on the level of apoptosis of the mononuclear cells of peripheral blood induced by cucurbit[n]urils (Figure 3). There were various effects of cucurbit[n]urils for T helpers and T cytotoxic lymphocytes. It was found that CB[6] and CB[7]—in all studied concentrations—barely affected the early or late apoptosis of CD8+ cytotoxic lymphocytes (Figure 3a,b). The addition of CB[8] did not result in a statistically significant increase in the percentage of apoptotic CD8+ cells. However, there was a tendency for the number of T cytotoxic cells to increase in the stage of early apoptosis compared to the “native” apoptosis of these cells in the samples freshly isolated from blood (*p* = 0.08) and the control (*p* = 0.06). CB[8] caused no effect on T cytotoxic cells in the stage of late apoptosis. The cucurbit[n]urils did not lead to a significant difference in the level of late apoptosis of CD4+ cells. It should be noted that the percentage of early apoptotic CD4+ cells cultured in the presence of all CB[n] increased compared to that in “native” apoptosis (Figure 3c). At the same time, the level of “native” apoptosis in peripheral blood did not demonstrate a statistically significant difference to apoptosis in the control culture. The difference in the level of early apoptosis of the T helpers between the control culture and the mononuclear culture with CB[n] was not significant. Nevertheless, there was a tendency (*p* = 0.08) to increase the amount of CD4+ T helper cells at the level of early apoptosis in the culture incubated with CB[8] compared to the control.

Therefore, the addition of CB[n] to a mononuclear cell culture can increase the level of early apoptosis of T lymphocytes. CB[8] had the greatest effect on the lymphocytes. Thus, the presence of CB[8] in a culture of mononuclear cells can increase the early apoptosis of T helpers more than 2.5 times compared to the level of “native” early apoptosis in peripheral blood.

### 3.3. Effect of Cucurbit[6,7,8]urils on the Hemolysis of RBC

**Figure 4:**
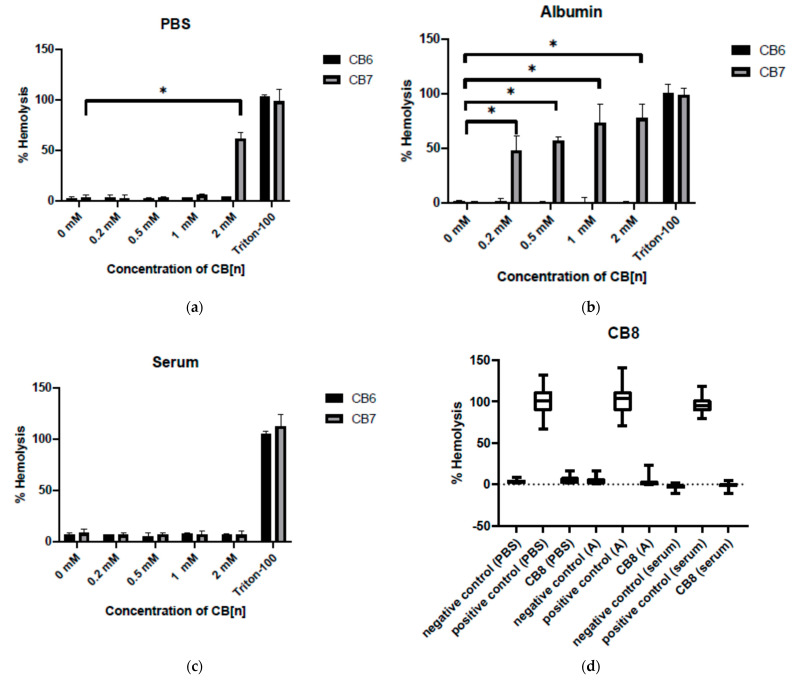
Effect of cucurbit[6,7,8]urils on the hemolysis of erythrocytes: (**a**) effect of CB[6] and CB[7] on the hemolysis of erythrocytes in PBS; (**b**) effect of CB[6] and CB[7] on the hemolysis of erythrocytes in albumin; (**c**) effect of CB[6] and CB[7] on the hemolysis of erythrocytes in autologous serum; (**d**) effect of CB[8] on the hemolysis of erythrocytes in different biologically relevant media. * Indicates a significant difference (*p* < 0.05) vs. the control. Data are presented as the median with the interquartile range (n = 8).

The level of hemolysis in three different media—PBS, albumin and autologous blood serum—was assessed (Figure 4). CB[6] and CB[8] were found to have no effect on erythrocyte hemolysis in any of the media. The various effects in the media were demonstrated by CB[7]. For example, hemolysis did not occur in the serum. However, the erythrocytes cultured with CB[7] at a concentration of 2 mM in PBS and at the concentrations of 0.2, 0.5, 1 and 2 mM in albumin underwent hemolysis. Consequently, CB[n] has almost no hemolytic activity in PBS, but they can have a hemolytic effect in complex biological relevant media close to physiological conditions.

## 4. Discussions

It is known that various nanoparticles can have toxic effects on cells. Cucurbiturils used as supramolecular containers for drug delivery or as a platform for designing various multicomponent systems in contrast to most of the studied nanoparticles. CB[n] has low toxicity for various types of cells [27,28,29,30]. In our research, we investigated the effect of CB[n] on blood cells. We demonstrated that CB[n] has no cytotoxicity effects up to 1 mM for PBMCs and the viability of PBMCs remained unchanged after exposure to CB[n].

Nevertheless, we found that CB[n] can increase the level of early apoptosis associated with the appearance of phosphatidylserine on the cell surface; even so, the integrity of the cell membrane was not damaged. In addition, it was shown that CB[7] can cause hemolysis in albumin, but not in PBS, excluding CB[7] at a concentration of 2 mM. A similar molecule, cyclodextrin (CyDs), also causes the hemolysis of erythrocytes. Cyclodextrin has been reported to induce shape changes of the membrane internalization type on erythrocytes. CyDs can release cholesterol from the erythrocyte membrane, thereby causing hemolysis [13]. It is known that CB[7] and other homologues (with the exception of CB[5] and CB[6]) can also bind cholesterol molecules [31], so the hemolytic effect of cucurbiturils may be similar to the effects of cyclodextrins.

Hemolysis with albumin is not as much associated with the toxic effects of cucurbiturils themselves as with the indirect interaction of CB[n] with medium components. Thus, it is known that CB[7] is capable of forming complexes with amino acids, peptides and proteins [21,22,23,24,25]. According to the literature, CB[7] and CB[8] can bind to albumin [32,33]. However, due to the low solubility of CB[8], we used it at lower concentrations than CB[7]. Consequently, CB[7] at the used concentration also interacts with the amino acid residues of albumin and, possibly, enhances its delivery to erythrocytes, which can cause cell death. We believe that the enhancement of hemolysis in the presence of CB[7] requires further study. Since the effect has not been observed in serum containing equivalent concentrations of albumin, it is possible that a similar result will only be observed in in vitro experimental studies. It should be noted that concentrations of CB[n] from 2 mM to 0.01 mM were used in this study and CB[n] seems to be safe for these cells at concentrations up to 0.3 mM. CB[n] in concentrations above 0.3 mM may not be required for drug delivery systems. The absence of such an effect of CB[6] may be due to the fact that CB[6] (in contrast to other CB[n]s) has a small cavity and other chemical and biological properties to effectively bind to biologically active molecules in the used medium.

Thus, the cucurbiturils studied by us do not damage cells directly, but they can affect the components of the cell microenvironment.
